# Dynamic changes in betanin content during the growing season of table beet: their interplay with abiotic factors

**DOI:** 10.18699/VJGB-22-05

**Published:** 2022-02

**Authors:** D.V. Sokolova

**Affiliations:** Federal Research Center the N.I. Vavilov All-Russian Institute of Plant Genetic Resources (VIR), St. Petersburg, Russia

**Keywords:** betanin, natural food coloring, dynamics, peel, flesh, Beta vulgaris L., environmental factors, бетанин, натуральный пищевой краситель, динамика, кожица, мякоть, Beta vulgaris L., факторы среды

## Abstract

The table beet, a widespread edible root crop known for its medicinal and antioxidant properties, early maturation, good shelf life, and high contents of bioactive compounds, vitamins and minerals, is used for the production of a natural red food dye. The relevance of this study is dictated by the lack of knowledge about the dynamic changes in the content of betanin during the growing season when developing table beet cultivars with a focus on pigment extraction. The article presents the results of a study of 29 red-colored table beet accessions from the collection of the N.I. Vavilov All-Russian Institute of Plant Genetic Resources (VIR). Dynamic changes in the content of the pigment during the growing season were observed on two beet accessions, cvs. ‘Russkaya odnosemyannaya’ and ‘Bordo odnosemyannaya’. Four pH versions of the buffer solution were tested, and the test results are presented. A buffer solution with pH 6.5 is recommended for research purposes. The amplitude of variability in the content of betanin in the peel (39.9–239.2 mg/100 g) and f lesh (14.4–127.5 mg/100 g) of beets was determined. It was conf irmed that the content of betanin in the peel exceeded that in the f lesh in all samples. A positive relationship between these indicators was revealed (r = 0.74, p ≤ 0.05). It was found that betanin accumulation did not occur in beet roots during the growing season. The pigment showed considerable f luctuations associated with abiotic environmental factors. Correlation analysis showed a signif icant positive relationship between air temperature and betanin content in the root f lesh (r = 0.32–0.31, p ≤ 0.05). A negative impact of environmental temperature on betanin content in the peel manifested itself on the third day (r = –0.34…–0.35, p ≤ 0.05). The negative response to precipitation was less expressed in cv. ‘Bordo odnosemyannaya’ due to the genotype’s more active metabolism and plasticity. Structural morphological features of the photosynthetic apparatus were described for the tested accessions, and their interrelations with the studied character were specif ied. Recommendations are given concerning the choice of a planting pattern and the timing of table beet harvesting for pigment extraction

## Abstract (ru)

Для производства натурального пищевого красителя красного цвета используется широко распространенная корнеплодная культура – столовая свекла, характеризующаяся лечебными, антиоксидантными свойствами, скороспелостью, длительной сохранностью корнеплодов, высоким содержанием биологически активных веществ, витаминов и минеральных элементов. Актуальность исследования продиктована недостатком знаний о динамических изменениях в содержании бетанина в течение вегетации при создании сортов свеклы, ориентированных на получение красителя. В работе приведены результаты изучения 29 красноокрашенных образцов коллекции столовой свеклы Всероссийского института генетических ресурсов растений им. Н.И. Вавилова. Наблюдение за динамическими изменениями содержания пигмента в процессе вегетации выполнено на двух образцах свеклы: ‘Русская односемянная’ и ‘Бордо односемянная’. Приведены результаты тестирования четырех вариантов pH буферного раствора. Рекомендован буферный раствор с pH 6.5. В результате исследования определена амплитуда изменчивости содержания бетанина в кожице (39.9–239.2 мг/100 г) и мякоти (14.4– 127.5 мг/100 г) корнеплодов столовой свеклы. Подтверждено, что содержание бетанина в кожице у всех образцов превышало его содержание в мякоти. Выявлена положительная взаимосвязь этих показателей (r = 0.74, p ≤ 0.05). Установлено, что в процессе вегетации значимой аккумуляции бетанина в корнеплодах не происходит. Показаны значительные колебания пигмента, сопряженные с абиотическими факторами среды. Определены корреляционные связи между температурой воздуха и бетанином в мякоти корнеплода (r = 0.32– 0.31, p ≤ 0.05). Отрицательный эффект температуры среды на бетанин в кожице проявлялся на третьи сутки (r = –0.34…–0.35, p ≤ 0.05). Негативная реакция на осадки была менее выражена у сорта ‘Бордо односемянная’, благодаря более активному метаболизму и пластичности генотипа. Описаны морфологические особенности строения фотосинтетического аппарата опытных образцов, отмечены взаимосвязи с изучаемым признаком. Даны рекомендации по выбору схемы посадки и сроков уборки урожая столовой свеклы для выделения красителя.

## Introduction

Red color shades for food production are mainly supplied by two groups of plant pigments: anthocyanins and betalains. Most flowering plants generate purple pigments, called anthocyanins (Yudina et al., 2021). The exceptions are representatives of several families in the order Caryophyllales: they synthesize betalains.

Betanin (betanidine-5-O-β-glucoside) is the main pigment (70–95 %) in the betalain group (von Elbe, 2001; Sawicki et al., 2016). It is a glycoside: its saccharide part is glucose, and its aglycone is betanidine. Betanin is a nontoxic compound that exhibits pronounced anti-inflammatory, anticarcinogenic and antioxidant properties, which is the reason why the interest in it is growing not only among food producers but also in the pharmaceutical and cosmetic sectors (Jiratanan, Liu, 2004; Tesoriere et al., 2004; Stintzing, Carle, 2007). An important advantage of betanin over anthocyanins is its stability in the pH range from 3 to 7, which makes it possible to use it as a color additive with both acidic and neutral media (Herbach et al., 2006). At the same time, a significant drawback of betanin is the degradation of the pigment upon heating – as a result of decarboxylation, the molecule of betanin loses its properties and is converted into neobetanin (Aztatzi-Rugerio et al., 2019).

The main sources of betanin are roots of table beet (Beta vulgaris L. ssp. vulgaris var. conditiva Alef.), fruits of prickly pear (Opuntia vulgaris Mill.), and red-colored forms of amaranth (Amaranthus L.) (Cai et al., 1998; Castellanos-Santiago, Yahia, 2008). The dominant place among them is occupied by the cultivated beet; the other sources of this pigment cannot compete with beet due to its high yield (50–60 t/ha), environmental plasticity, and high produce of betanin (Stintzing et al., 2000; Sokolova, 2018).

The history of the use of betanin for coloring food products began in the early 20th century: the pigment was added to pastries, dry mixes, and dairy and meat products. The dye is known to be used in the form of a juice concentrate and a dry powder obtained through freeze- or spray-drying (Nemzer et al., 2011). Publications analyzing the effect of the processing of beet raw materials on the pigment content include a mandatory peeling of the beetroot in their guidelines (Azeredo et al., 2009; Burak, Zavaley, 2020), which is certainly necessary for the preparation of juices, for baby food and healthy diets. For dye production, however, the unpeeled roots undergo blanching (Frolov, Chizhik, 1997).

Generation and accumulation of betalain pigments, including betanin, in table beet plants are considered to be a dynamic process that depends not only on the specific genotype and the phase of ontogenesis but also on various environmental factors, the maturity of roots, their size, agricultural practice, and soil fertility (Mglinets, Osipova, 2010; Vulić et al., 2013). Betalain stability is affected by numerous external and internal factors: temperature, acidity, presence/absence of light, oxygen, enzymes, nitrogen, metal cations, and the degree of glycosylation and acylation. Such data, in most cases, were obtained when studying the pigment extracted from mature roots (Saguy et al., 1978; Saguy, 1979; Schliemann, Strack, 1998; Herbach et al., 2006).

The objective of this study was to trace the dynamic changes in the pigment content during the growing season separately for the peel and flesh of table beet roots and measure the effect size of environmental factors.

## Materials and methods

A set of 29 accessions of red-colored table beet (Beta vulgaris L. var. conditiva Alef.) from the collection of the N.I. Vavilov All-Russian Institute of Plant Genetic Resources (VIR) served as the material for the research. Dynamic changes in betanin content during the growing season were observed on two beet accessions: cvs. ‘Russkaya odnosemyannaya’ (k- 3698) and ‘Bordo odnosemyannaya’ (k-3151), identified as the most promising during the screening and ecogeographic study of the VIR collection in 2008–2018 (Sokolova, 2019; Sokolova, Solovieva, 2019).

Field experiments were conducted according to a unified methodology (Burenin, 1989) in 2020 at Pushkin and Pavlovsk Laboratories of VIR (Pushkin, St. Petersburg, Russia). Soils in Pushkin are mainly sod podzols and sandy loams. The accessions were cultivated under natural conditions, without fertilizers or pesticides. The area of record plots was 24 m2. Sowing was done manually on May 28, and harvesting on September 24. The distance between rows was 70 cm, with 6–8 cm between plants in a row.

Observations of the weather conditions during the growing season were conducted at the VIR Hydrometeorological Station. Weather conditions in 2020 were favorable for table beet cultivation, with moderate air temperatures throughout the entire growing period. The sum of active temperatures (˃ +10 °C) from May 20 to September 24 was 2009 °C; the precipitation amount for the same period was 290 mm, i. e., 36 mm lower than the mean values for many years.

For the analysis of dynamic changes in betanin content, roots of cvs. ‘Russkaya odnosemyannaya’ (k-3698) and ‘Bordo odnosemyannaya’ (k-3151) were collected twice a week from July 13 to September 24, 2020. The pigment content was analyzed using the peel (cut with a knife at a depth of 1–2 mm) and the flesh of ten roots separately. All measurements were taken within 3 hours after removing the roots from the soil. The juice was squeezed from the test samples using a Bork JU CUP 21085 WT juicer (Germany) and filtered through a membrane filter (0.45 microns). The filtered juice (1–1.5 g per sample) was weighed and diluted with a phosphate buffer (pH 6.5) up to the mark of 100 ml. Betanin content was measured spectrophotometrically at a wavelength of 538 nm. Measurements at 600 nm were used to correct for impurities. The absorption peak at 538 nm reflects the structure and is used to analyze betanin without isolating specific pigments. The filtrate from the roots was studied on a Shimadzu UV-1800 double-beam spectrophotometer (Japan). Betanin concentrations (CB) were measured according to Nilsson (1970) as follows:

**Form 1.**
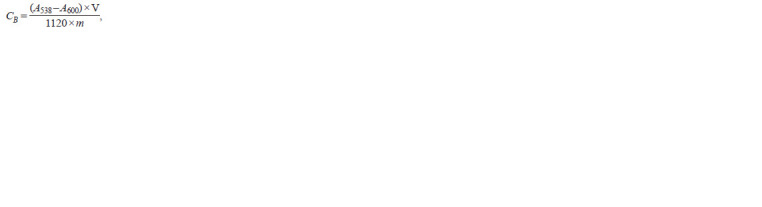
Formula 1.

where: А538 is the optical density at 538 nm; А600 is the optical density at 600 nm; V is the dilution ratio; m is the mass of the sample, g; 1120 is the specific absorption of 1 betanin solution in a 1 cm cuvette.

The data were statistically processed using the Excel and Statistica 8.0 software. The variability in the structure of relationships among characters was assessed using factor analysis. Factor loadings were calculated using the principal component method. The values of the Pearson correlation coefficient at r < 0.3 were considered as weak, 0.3 < r <0.5 as moderate, 0.5 < r < 0.7 as conspicuous, 0.7 <r < 0.9 as strong, and r > 0.9 as very strong.

## Results and discussion

**Fig. 1.**
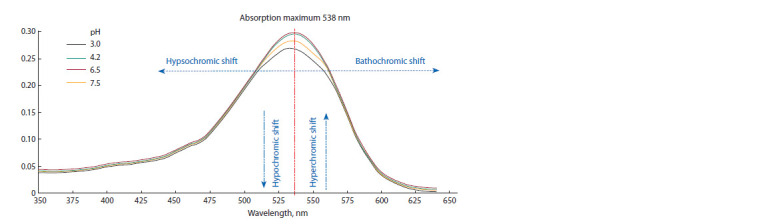
Betacyanin solution spectra of Beta vulgaris L. at different pH of the buffer solution (cv. ‘Russkaya odnosemyannaya’, k-3698).

Betanin has the maximum light absorption in the visible spectrum range at wavelengths from 535 to 540 nm. Four pH versions were tested to select the optimal pH of the buffer solution using the example of cv. ‘Russkaya odnosemyannaya’ (k-3698, Russia) (Fig. 1). The spectra of betanin solutions with the same initial concentration manifested changes in the pH range from 3 to 7.5: there was a slight hypochromic shift at pH 3 and 7.5. No displacement of the absorption maximum was observed. A phosphate buffer solution with a pH of 6.5 was used in this study.

**Fig. 2.**
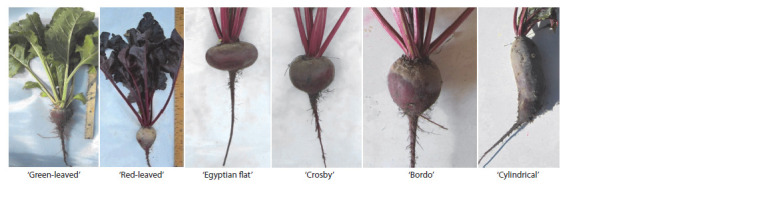
Cultivar types of table beet accessions in the experiment.

The phenotypic diversity of table beet is usually grouped into cultivar types. Such grouping is based on the similarity of morphological parameters (Fig. 2). This study included table beet accessions of six cultivar types.

The group of accessions representing the Crosby cultivar type, characterized by the rounded shape of the taproot, demonstrated the highest yield. The testing of 29 red-colored table beet accessions showed that on September 24 the average yield was 16.5 kg/10 m2 (Table 1). The yield indicator varied significantly depending on the genotype of an accession ( p <0.05). The average weight of one taproot was 127.9 g. Variation of this indicator within each cultivar was insignificant (coefficient of variation: CV < 33.3 %), attesting to the alignment of the populations. Cv. ‘Russkaya odnosemyannaya’ (k-3698) and the local cultivar population from Kazakhstan (k-3885) were the exceptions: their coefficients of variation were 35.1 and 40.1 %, respectively.

**Table 1.**
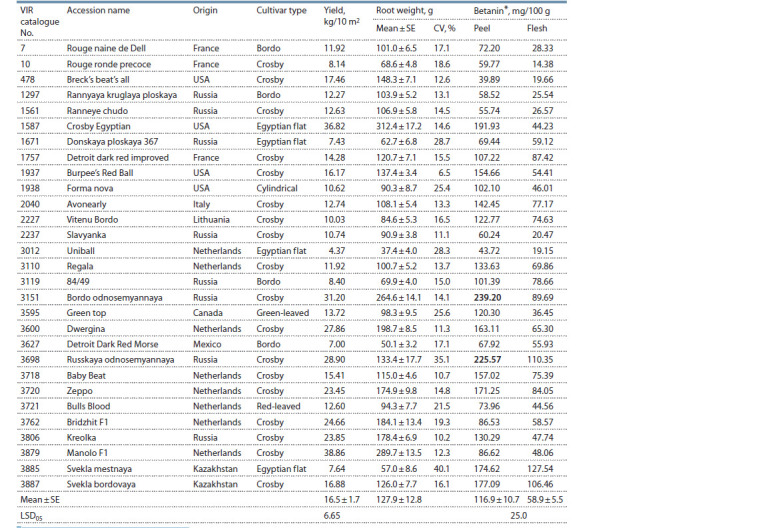
Yield and betanin content of the 29 tested table beet accessions * Values are obtained from 10 roots per accession.

The betanin content at the time of harvesting varied significantly, and the range of variations was wide. The average value was 116.9 mg/100 g in the peel, and 58.9 mg/100 g in the root flesh. The pigment content was observed to depend on the morphological type of the root and the intensity of its color. Thus, our earlier conclusions about the preference of the rounded or roundish oval root shape for breeding for high betanin content (Sokolova, Solovieva, 2019) were confirmed. It is worth mentioning that there were exceptions among the flattish round accessions, which, as a rule, represented early maturing forms with relatively low betanin content (Sokolova, 2019). For example, the cultivar of American origin ‘Crosby Egyptian’ (k-1587) and the local cultivar from Kazakhstan ‘Svekla mestnaya’ (k-3885) also demonstrated high levels of betanin content in their peel: 191.93 and 174.62 mg/100 g, respectively.

The highest betanin content in the peel of the root was recorded in the accessions of domestic origin: ‘Bordo odnosemyannaya’ (k-3151) and ‘Russkaya odnosemyannaya’ (k- 3698) – 239.20 mg/100 g and 225.57 mg/100 g, respectively. These genotypes are of interest for further breeding practice aimed at increasing the pigment content.

**Fig. 3.**
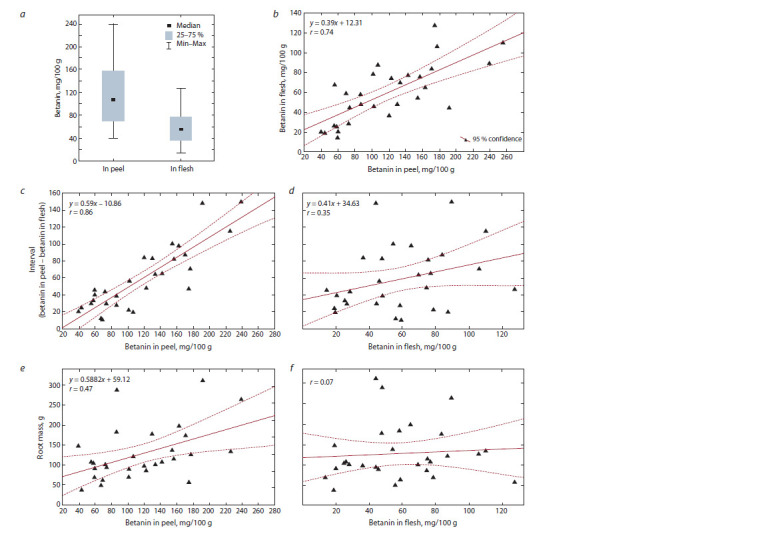
Mean betanin content in the peel and f lesh of table beet roots (a). Linear correlation analysis: correlation between betanin content in the peel and that in the f lesh of table beet (b); betanin in the peel and the ratio of the two parameters (betanin in the peel to betanin in the f lesh) (c); betanin in the f lesh and the ratio of the two parameters (d ); betanin in the peel and the root weight (e); betanin in the f lesh and the root weight (f ).

Separate analyses of betanin content in the peel and flesh of beet roots revealed statistically significant differences. The pigment content in the peel of all the studied accessions exceeded this parameter in the flesh (Fig. 3, a). The smallest difference was observed in ‘Donskaya ploskaya 367’ (k-1671): 10.3 mg/100 g. The greatest difference was demonstrated by cv. ‘Bordo odnosemyannaya’: 149.5 mg/100 g, with the ratio 73 % of betanin in the peel to 27 % in the root flesh. Correlation analysis showed the presence of a significant positive relationship between the pigment contents in the peel and flesh (r = 0.74) (see Fig. 3, b). At the same time, when betanin content in the peel was higher, the span between the parameters also increased (r = 0.86) (see Fig. 3, c). Unlike betanin in the peel, no significant correlation was found between the pigment content in the flesh and the variations between the parameters, which attested to its higher stability in the flesh during the growing season (see Fig. 3, d ). Thus, an increase in the total betanin content depends, first of all, on the content of the pigment in the peel.

No significant correlation (r ˃ 0.5) was found between the root weight and the betanin content in the peel and flesh (see Fig. 3, e, f ).

**Fig. 4.**
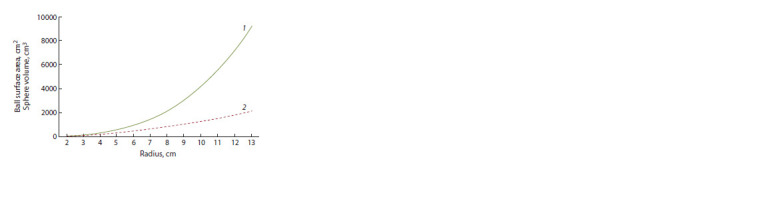
Dependence of the volume (1) and surface area (2) of a sphere on its radius.

It is important for the production of dye from beet roots that the pigment content in the total biomass is high. If the shape of the table beet root is schematically regarded as a sphere, it is possible to calculate how the ratio between its volume and surface area will change depending on the radius. The diagram (Fig. 4) demonstrates that the increase in the volume of the sphere has a cubic dependence, while the expansion of the surface area has a quadratic one. Extrapolating the theoretical layout to the object of this study, it can be argued that, assuming that the thickness of the peel is constant in mature, ready-to-harvest beet roots, the portion of the flesh significantly exceeds that of the peel, which will ultimately have a negative effect on the output of the pigment. Meanwhile, the share of the peel in smaller table beet roots tends to be higher than that in large ones. It can be therefore assumed that the total yield of betanin extracted from smaller roots will be higher, provided that the pigment content is high at the time of harvesting.

**Fig. 5.**
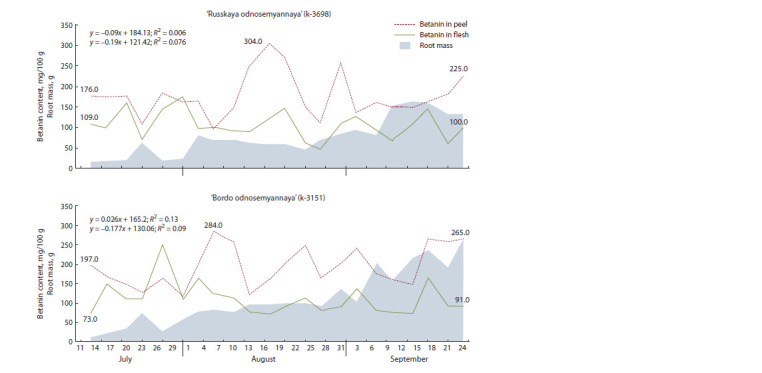
Dynamic changes in betanin content and root weight in the table beet cultivars ‘Russkaya odnosemyannaya’ and ‘Bordo odnosemyannaya

To understand the dynamics of root growth and pigment accumulation separately in the peel and flesh, we conducted a comparative analysis of two promising table beet accessions: ‘Russkaya odnosemyannaya’ (k-3698) and ‘Bordo odnosemyannaya’ (k-3151). The material for the analysis was collected 22 times at regular intervals from the moment when the roots exceeded the weight of 10 g. No significant accumulation of betanin in the peel or flesh of both cultivars was recorded during the growing season (Fig. 5), which confirmed the results of the earlier ecogeographic study (Sokolova, 2019). At the same time, the range of variations in individual periods of development was statistically significant ( p < 0.05). The dynamics had a wavelike pattern and was fairly synchronous for both cultivars, both in the peel and in the root flesh. It can be noted that abrupt upward movements of betanin content and root weight increases were often directed differently. Besides, the pigment content in the flesh changed more conservatively than in the peel.

The pathway of betanin biosynthesis is known to be quite flexible and strongly influenced by exogenous factors (Tzin, Galili, 2010; Cabanes et al., 2014; Sakuta, 2014; Esatbeyoglu et al., 2015). An excess in the content of betanin in the flesh over its content in the peel for both cultivars was observed only once during the entire growing season (the period from July 27 to July 31). Concurrently, its maximum in the flesh for the entire growing season was recorded. Abundant continuous rainfall that settled from July 21, together with mean daily air temperatures above +15 °C, caused inhibition of root development, which most likely led to an increase in betanin content in the root flesh.

Unlike the pigment content, an increase in the root biomass positively correlated with the duration of the growing season (R2 = 0.76–0.86).

**Fig. 6.**
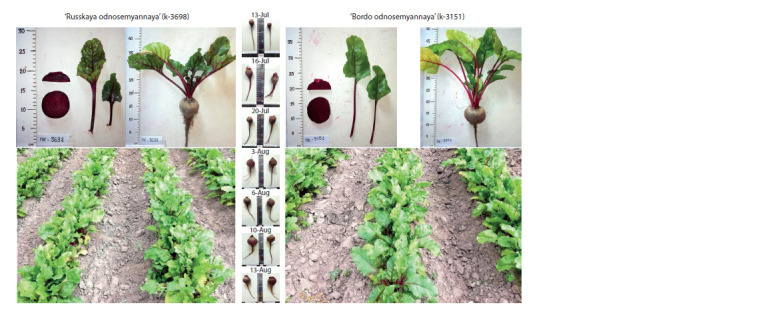
Anatomical and morphological structure and appearance of f ield crops of experimental table beet accessions ‘Russkaya odnosemyannaya’ (k- 3698) and ‘Bordo odnosemyannaya’ (k-3151).

The activity of photosynthesis and, as a consequence, plant metabolism is associated with the surface area of the plant’s leaf biomass. The plants of cv. ‘Russkaya odnosemyannaya’ had small, pigmented and slightly wavy leaf blades with shorter petioles (Table 2, Fig. 6). The number of leaves per plant was less than that of cv. ‘Bordo odnosemyannaya’. At the time of harvesting, the leaf surface area of ‘Russkaya odnosemyannaya’ was 494.2 cm2, which was almost twice less than that of ‘Bordo odnosemyannaya’. These characteristics are important for understanding the differences in the cultivars’ response to abiotic environmental factors.

**Table 2.**
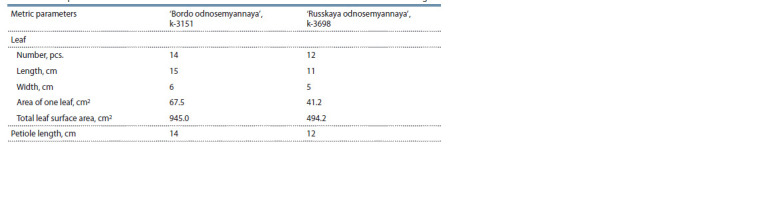
Metric parameters of the leaf surface area of the tested table beet accessions at the time of harvesting

**Fig. 7.**
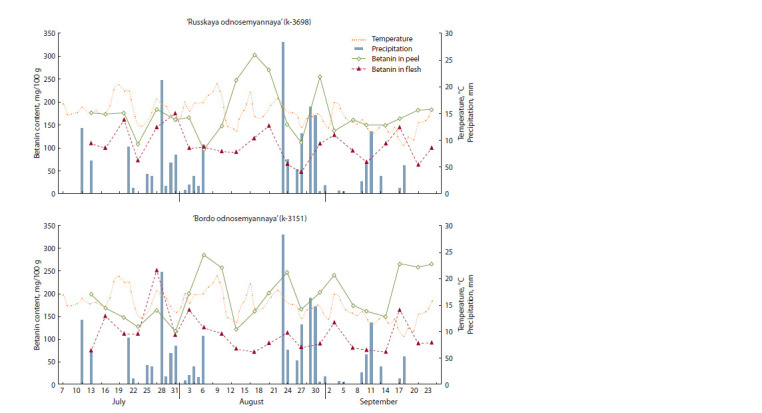
Dynamic changes in the characteristics of weather conditions and the content of betanin in the table beet cultivars ‘Russkaya odnosemyannaya’ and ‘Bordo odnosemyannaya’.

Mean daily air temperature and rainfall are two of the most influential and constantly fluctuating environmental factors. Table beet is a fairly adaptive crop: it is capable of producing harvests both in the southern regions of Russia and under the conditions of the north (Burenin et al., 2013). It was this quality that made the beet crop widespread. The study has shown that the high plasticity of the genotype is a valuable property that allows the crop to safely endure unfavorable periods. Figure 7 presents the dynamics of the pigment content with varying rainfall and air temperature values. The growing season in 2020 was characterized by two stressful periods with complete absence of precipitation: from July 14 to 20, and from August 7 to 22. There were also two periods with prolonged rainfall: from July 21 to August 6, and from August 23 to September 1. Significant precipitation amounts were registered within one day on July 28 and August 23 (21.2 and 28.2 mm, respectively). The same weather held on for 9 days, although with less intensive rains. It negatively affected betanin content in the table beet peel and flesh. The response of both cultivars in terms of their pigment content was practically similar, but there were some differences. The graph also shows a lag of 7 to 10 days in the response of ‘Russkaya odnosemyannaya’ (see Fig. 7).

To clarify the relationships between the studied parameters, a correlation analysis of the cultivars was conducted in dynamics (3-day and 6-day shifts of environmental parameters) (Table 3). Both cultivars demonstrated a moderate negative correlation between root weight and air temperature, which persisted for 6 days. Higher temperatures increased the transpiration of plants, arresting the growth of roots and raising the pigment content in the root flesh (r = 0.32–0.31). Three days later, the effect of an increase in the pigment concentration in response to temperature was no longer manifested.

**Table 3.**
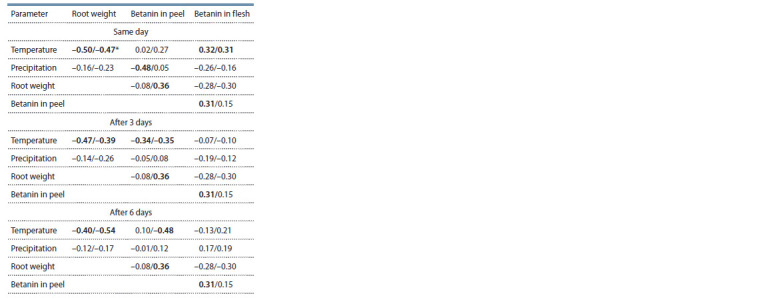
The correlation matrix for the indicators of the table beet cultivars * ‘Russkaya odnosemyannaya’/‘Bordo odnosemyannaya’.

Betanin content in the peel also negatively correlated with air temperature increases: this effect did not appear immediately, but on the third day. This correlation was retained by cv. ‘Bordo odnosemyannaya’ until the 6th day (r = –0.48).

Precipitation in general was characterized by weak negative correlations with all the studied characters. Rainfall had a weak correlation with the pigment content (r < 0.3). Betanin biosynthesis in the peel of ‘Russkaya odnosemyannaya’ reacted negatively to precipitation (r = –0.48). Three days later, however, the plants adapted and this correlation was not observed. The differences in the response of the cultivars may be explained by the difference in the photosynthetic surface area of the genotypes.

**Fig. 8.**
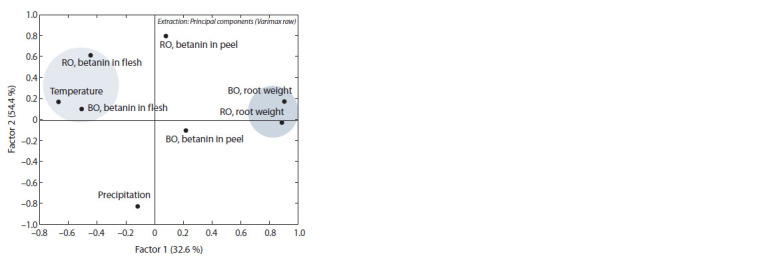
The scatter chart showing the contribution of principal components in the factor analysis to generalized variance. RO – ‘Russkaya odnosemyannaya’ , BO – ‘Bordo odnosemyannaya’ .

Research into the structure of relationships among the studied parameters (PCA) disclosed two unbalanced factors (Fig. 8). The components of Factors 1 and 2 together explain 87 % of the total variance: 32.6 and 54.4 %, respectively. Factor 1 included the main parameter of productivity – the weight of one root. Factor 2 combined the indicators “temperature” and “betanin in flesh”, confirming the results of the correlation analysis. The indicators within Factor 2 had the closest relationship, i. e., they accounted for most of the variance. At the same time, other variables also had a fairly high variance, which confirms the significant contribution of each of them. Thus, we can conclude that the process of betanin biosynthesis in table beet is extremely flexible and strongly affected by environmental factors.

## Conclusion

The results of the study make it possible to conclude that during the growing season there was no accumulation of betanin in table beet roots. The pigment content was associated primarily with the original genotype of an accession. Mean values of betanin content in the studied accessions were: 116.9 mg/100 g in the peel, and 58.9 mg/100 g in the flesh. The pigment content in the peel exceeded its content in the flesh in all accessions. With a positive correlation between these indicators (r = 0.74), an increase in the span of the ratio between them depended precisely on betanin in the peel (r = 0.86).

The pigment content was subject to significant fluctuations during the growing season. The activity of metabolic processes that depended on the photosynthetic surface of plants was of great importance for the response of table beet genotypes to various environmental factors.

The pigment biosynthesis process was extremely sensitive to weather conditions, especially air temperature. An increase in betanin content in the peel in response to an increase in air temperature manifested itself on the third day. An increase in the mass of one root negatively correlated with the content of betanin in the root flesh, which, in its turn, was closely associated with air temperature.

Table beet cultivars with a round or flattish round shape of their root are the most suitable for breeding targeted at higher betanin content. Smaller beet roots are better suited for obtaining the maximum betanin yield. Denser planting patterns or earlier harvesting are recommended for their cultivation, as well as optimal timing. When choosing a specific date for harvesting, it is necessary to focus on the characteristics of weather conditions. The domestic table beet cultivars ‘Bordo odnosemyannaya’ (k-3151) and ‘Russkaya odnosemyannaya’ (k-3698) are recommended for extracting the betanin dye, because under favorable conditions they are capable of yielding high amounts of the pigment.

## Conflict of interest

The authors declare no conflict of interest.
